# Probing nearby molecular vibrations with lanthanide-doped nanocrystals†

**DOI:** 10.1039/d3nr02997b

**Published:** 2023-10-03

**Authors:** Mark J. J. Mangnus, Vincent R. M. Benning, Bettina Baumgartner, P. Tim Prins, Thomas P. van Swieten, Ayla J. H. Dekker, Alfons van Blaaderen, Bert M. Weckhuysen, Andries Meijerink, Freddy T. Rabouw

**Affiliations:** a Inorganic Chemistry and Catalysis group, Debye Institute for Nanomaterials Science and Institute for Sustainable and Circular Chemistry, Utrecht University Universiteitsweg 99 3584 CG Utrecht The Netherlands f.t.rabouw@uu.nl; b Soft Condensed Matter group, Debye Institute for Nanomaterials Science, Utrecht University Princetonplein 1 3584 CC Utrecht The Netherlands; c Condensed Matter and Interfaces group, Debye Institute for Nanomaterials Science, Utrecht University Princetonplein 1 3584 CC Utrecht The Netherlands

## Abstract

The photoluminescence (PL) of lanthanide-doped nanocrystals can be quenched by energy transfer to vibrations of molecules located within a few nanometers from the dopants. Such short-range electronic-to-vibrational energy transfer (EVET) is often undesired as it reduces the photoluminescence efficiency. On the other hand, EVET may be exploited to extract information about molecular vibrations in the local environment of the nanocrystals. Here, we investigate the influence of solvent and gas environments on the PL properties of NaYF_4_:Er^3+^,Yb^3+^ upconversion nanocrystals. We relate changes in the PL spectrum and excited-state lifetimes in different solvents and their deuterated analogues to quenching of specific lanthanide levels by EVET to molecular vibrations. Similar but weaker changes are induced when we expose a film of nanocrystals to a gas environment with different amounts of H_2_O or D_2_O vapor. Quenching of green- and red-emitting levels of Er^3+^ can be explained in terms of EVET-mediated quenching that involves molecular vibrations with energies resonant with the gap between the energy levels of the lanthanide. Quenching of the near-infrared-emitting level is more complex and may involve EVET to combination-vibrations or defect-mediated quenching. EVET-mediated quenching holds promise as a mechanism to probe the local chemical environment—both for nanocrystals dispersed in a liquid and for nanocrystals exposed to gaseous molecules that adsorb onto the nanocrystal surface.

## Abstract 2

Energy transfer to molecular vibrations can quench the photoluminescence of lanthanide-doped nanocrystals. We investigate the potential of this phenomenon for chemical sensing of liquid- and gas-phase molecules.

## Graphical abstract

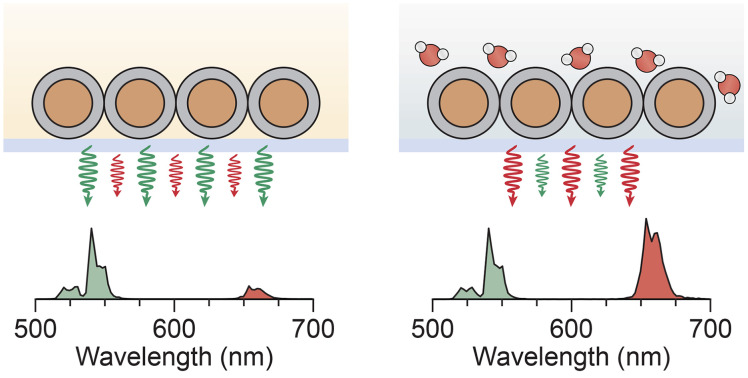

## Introduction

Photoluminescence (PL) from lanthanide dopants in nanocrystals (NCs) is often less efficient than from dopants in bulk analogs.^1–5^ Energy transfer from an electronically excited lanthanide ion to high-energy vibrational modes of molecules at the NC surface or in the immediate environment competes with photon emission and therefore lowers the PL quantum yield (QY).^1,5–13^ This electronic-to-vibrational energy transfer (EVET) is a type of Förster resonance energy transfer, which is short-ranged (order of nanometers) and requires that the energy gap between emitting levels of the donor excited state matches the acceptor mode energy of a nearby molecular vibration.^9,11,14–16^ EVET is undesired for most applications but can be prevented by growing a protective shell of undoped material around the doped core.^2,5,6,8,11–13,17,18^ Alternatively, control over EVET rates from different excited states of the lanthanide means control over their respective QYs, which is an interesting strategy to tune the spectral distribution of the PL.^6^

Previous works have shown that the rate of EVET varies strongly for lanthanide-doped NCs dispersed in media with different vibrational energies, such as H_2_O and D_2_O (with vibrational energies *ħω* of ∼3600 cm^−1^ and ∼2600 cm^−1^, for the O–H and O–D stretching vibrations),^9,15,16^ or aliphatic and aromatic hydrocarbons (*ħω* of ∼2900 cm^−1^ and 3050 cm^−1^).^11,19^ Interestingly, as the vibrational energies of molecules are fingerprints of their chemical identity, the rate of EVET should provide information about the local chemical environment of lanthanide-doped NCs.

Here, we investigate the potential of lanthanide-doped NCs to probe nearby molecules with different molecular vibrations. We show that the PL spectrum of lanthanide-doped NCs changes when the NCs are dispersed in different solvents and their deuterated analogues. The PL lifetime exhibits a similar sensitivity, which in some cases can be understood in terms of a simple EVET resonance, while a combination of molecule vibrations is likely responsible in other cases. A second set of experiments with a dry NC film shows sensitivity to gas-phase molecules: EVET to surface-adsorbed gas molecules is sufficiently strong to produce a clear response of the NC emission spectrum. Our experiments highlight that lanthanide-doped NCs can indirectly probe molecular vibrations with fingerprints in the mid-infrared region by giving a response in visible, in the form of a change in the lanthanide PL spectrum and lifetimes. Our findings pave the way for applications in the fields of *e.g.*, catalysis^20^ and biosensing,^21–23^ where the PL spectrum of lanthanide-doped NCs responds to both temperature^24–26^ and the diffusion, formation, or conversion of molecules in the NC's local environment.

## Experimental methods

### Synthesis of oleic-acid-coated NaYF_4_:Er^3+^,Yb^3+^ NCs

Spherical oleic-acid-coated NaYF_4_:Er^3+^(2%),Yb^3+^(18%) core-only NCs were synthesized using the procedure reported in ref. 24. The NCs were stored in cyclohexane. Part of the NCs was transferred to other nonpolar solvents by precipitating with two volume equivalents of ethanol and redispersing in both isotopologues of cyclohexane and toluene. The other part of the NCs was used to synthesize silica-capped NCs. All chemicals were used without further purification.

### Synthesis of NaYF_4_:Er^3+^,Yb^3+^@SiO_2_ nanoparticles

Silica-coated NaYF_4_:Er^3+^(2%),Yb^3+^(18%) nanoparticles were prepared by silica shell growth around the core-only NCs using a microemulsion method.^24,27^ The NCs were stored in ethanol. The NCs were transferred to other polar solvents by centrifugation and redispersing. All chemicals were used without further purification.

### Photoluminescence measurements

Upconversion PL spectra of solution-dispersed nanoparticles were measured using an Edinburgh Instruments FLS920 fluorescence spectrometer equipped with a Hamamatsu R928 photomultiplier tube. Samples were excited using a 980 nm laser operated in CW-mode (Coherent OBIS LX, 150 mW). To filter out excitation light in the detection path, a Thorlabs FESH0900 900 nm short-pass filter was used. For time-resolved PL measurements of dispersed NCs, samples were excited by direct pulsed excitation at 515, 650 or 980 nm with an Ekspla NT342B laser at a repetition rate of 10 Hz. The PL at 536 and 660 nm was first filtered using a 516- and 654-nm long-pass filter, respectively, followed by detection using a Triax 550 monochromator and Hamamatsu H7422-02 photomultiplier tube, coupled to a PicoQuant TimeHarp 260 photon counting module. The PL at 1000 nm was detected using a Thorlabs FELH1000 long-pass filter and an avalanche photodiode (APD; Micro Photonics Devices PDM).

Layers of NCs were prepared by drop-casting 50 μL of the NC solution onto a glass coverslip, cleaned using a Diener Zepto low-pressure plasma cleaner, operated at 0.2–0.4 mbar and at the maximum power setting. Optical measurements on as-prepared layers of NCs were conducted on a Nikon Ti–U inverted microscope body, on which the sample was placed inside an air-tight cell (a Linkam THMS600 microscopy stage with a home-built glass lid to enable shorter working distances). Samples were purged with dry nitrogen for at least 30 minutes before starting the measurements. The gas flow through the cell was controlled by combining a flow of dry nitrogen gas (250 mL min^−1^) with a flow (0 or 50 mL min^−1^) of dry nitrogen gas bubbled through deionized H_2_O or D_2_O. Flow rates were controlled using mass flow controllers. The dry nitrogen gas flow was purified from moist and organic impurities using combined SGT-F0205 and SGT-F0103 filter columns. Assuming that the concentration of water vapor in the bubbler is governed by the saturation pressure of water (*p*_0_ = 31.7 mbar at room temperature; ref. 28), the partial water pressure in the humid water flow with *p*/*p*_0_ = 0.17 equals 5 mbar. A 980 nm laser (Coherent OBIS LX, 150 mW), operated in block-pulsed mode with a repetition rate of 250 Hz and a 50% duty cycle using an Aim-TTi TGA1244 waveform generator, was directed to the sample using a 900 nm short-pass dichroic mirror (Thorlabs DMSP900R), and defocused with a microscope objective (Nikon CFI S Plan Fluor ELWD 60XC) to create a spot with a diameter of ∼70 μm. The excitation powers were 5–10 W cm^−2^. Photoluminescence from the sample was collected using the same objective, guided through a 900 nm short-pass filter (Thorlabs FESH0900), and directed toward the detectors. Using a 50/50 beam splitter, 50 percent of the PL was focused onto the entrance slit of a spectrometer (Andor Kymera 193i) equipped with a reflective diffraction grating (150 lines per mm, blazed at 500 nm), coupled to an electron-multiplying CCD-camera (Andor iXon Ultra 888) operated at a frame rate of 2 Hz. The other 50 percent was divided among two avalanche photodiode detectors (Micro Photonic Devices PDM) that collected the green and red PL using a band-pass filter (Chroma ET535/70 m) and a combination of short- and long-pass filters (Thorlabs FESH0700/Thorlabs FELH0600). Time-tagged time-resolved data were obtained using a quTools quTAG time-to-digital converter, which was connected to both APDs and the laser driver and communicated all photon detection events and laser pulses.

### Infrared spectroscopy

The setup used for attenuated total reflectance Fourier-transform infrared (ATR-FTIR) spectroscopy has previously been described in ref. 29 and 30. ATR crystals (20 × 10 × 0.5 mm, 45°) cut from a double side polished Si wafer were used, with a depth of penetration *d*_p_ = 0.52 μm (*ṽ* = 1600 cm^−1^) and effective pathlength of *d*_e∥_ = 0.64 μm *d*_e⊥_ = 0.32 μm for the two polarization directions, yielding a total effective pathlength of (*d*_e∥_ + *d*_e⊥_)*N*/2 = 9.65 μm with *N* = 20. ATR-FTIR spectra were recorded using a Spectrum Three FTIR spectrometer (PerkinElmer) equipped with a N_2_-cooled mercury cadmium telluride (MCT) detector. 128 scans (1 spectrum per min) were averaged. The temperature of the cell was kept steady at 20 °C. The preparation of layers of NCs was analogous to the sample preparation for optical spectroscopy, except that a clean silicon ATR crystal was used as a substrate. The coated ATR crystal was placed inside an air-tight cell and the gas flow through the cell was controlled by combining a flow of dry nitrogen gas (Linde, 5.0) with a flow of dry nitrogen gas bubbled through deionized H_2_O or D_2_O. The total flow was kept constant at 100 mL min^−1^. Samples were again purged with dry nitrogen for at least 30 min before starting the measurements. Water vapor concentrations were determined by transmission IR spectroscopy in a 10 cm transmission cell with ZnSe windows. Concentrations were obtained from the band areas using reference spectra of 1 ppm m^−1^ water from the PNNL database.^31^ The application sequence started with pure N_2_ flushing for 10 min. Subsequently, the partial pressure was increased/decreased and kept constant for 3 min at each step to reach equilibrium.

## Results

### Photoluminescence of upconversion nanocrystals in different chemical environments

**Fig. 1:**
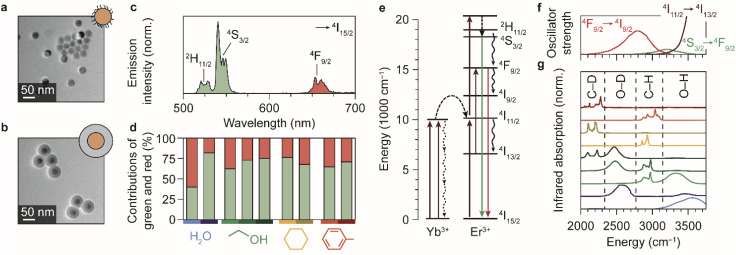
Upconversion luminescence from lanthanide-doped nanocrystals in different chemical environments. (a) Transmission electron microscopy images of the oleic-acid-coated NaYF_4_:Er^3+^(2%),Yb^3+^(18%) NCs. (b) Same, but for the silica-coated NCs. The cores are the regions with highest contrast. (c) Upconversion PL spectrum of the oleic-acid-coated NaYF_4_:Er^3+^(2%),Yb^3+^(18%) NCs, dispersed in cyclohexane. (d) Relative contributions of the green and red PL in different solvents. Measurements are done in H_2_O, ethanol, cyclohexane, and toluene under continuous-wave illumination at 10^−1^–10^0^ W cm^−2^. Light shades of blue, green, yellow, and red represent the normal solvents; darker shades represent the deuterated isotopologues D_2_O, ethanol-d1 (CH_3_CH_2_OD), ethanol-d6 (CD_3_CD_2_OD), cyclohexane-d12, and toluene-d8. Note that for ethanol, three different shades of green are used to represent the normal, O-d-substituted and fully deuterated isotopologues. (e) Upconversion in Yb^3+^–Er^3+^ co-doped NCs proceeds by excitation of Yb^3+^ sensitizers and subsequent multi-step energy transfer to Er^3+^ ions, which emit in the green and red. Important quenching processes are indicated by black curvy arrows. (f) Spectra of the electronic transitions in Er^3+^ that can act as EVET donors (ESI,† section S2). (g) Infrared absorption spectra of solvents molecules, reproduced from ref. 38 and 39. Characteristic vibrational energies of relevant chemical groups are highlighted within the dashed regions. Colors are the same as in panel d.

We synthesized spherical core-only β-NaYF_4_:Er^3+^(2%),Yb^3+^(18%) NCs with a diameter of 28.5 ± 1.8 nm (Fig. 1a).^24^ The NCs are capped with oleic acid ligands and can thus be readily dispersed in non-polar media. We used these cores to also synthesize silica-coated NaYF_4_ particles,^24,27^ with a shell thickness of 14.2 ± 1.2 nm (Fig. 1b), which are dispersible in polar solvents. Er^3+^–Yb^3+^ co-doped NCs exhibit near-infrared-to-visible photon upconversion: Yb^3+^ sensitizer ions absorb light at approximately 980 nm and transfer multiple excitations to Er^3+^ to populate visible-emitting energy levels (Fig. 1e).^15,32–34^ As an example, the upconversion PL spectrum of oleic-acid-coated NCs dispersed in cyclohexane (Fig. 1c) shows the resulting green and red emission lines originating from the ^2^H_11/2_/^4^S_3/2_ and ^4^F_9/2_ levels, respectively.

To investigate the impact of solvent molecules on the upconversion PL, we transferred the oleic-acid-coated and silica-coated NCs to a range of solvents, as well as to deuterium-substituted isotopologues of these solvents: H_2_O, D_2_O, CH_3_CH_2_OH, CH_3_CH_2_OD, CD_3_CD_2_OD, C_6_H_12_, C_6_D_12_, C_6_H_5_CH_3_, and C_6_D_5_CD_3_. Substitution of hydrogen atoms by the heavier deuterium isotope reduces the vibrational energies of the solvent molecules approximately by a factor 
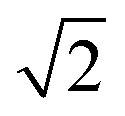
. We measured the upconversion PL of the different samples upon excitation with a 980 nm laser operated at a constant estimated power of 10^−1^–10^0^ W cm^−2^ to exclude a power-dependent effect.^10,34,35^ The relative contributions of green and red PL to the total upconversion PL differs substantially for NCs in deuterated and non-deuterated solvents (Fig. 1d; see also the ESI,† section S1). Differences in the relative contributions of the green and red upconversion PL are thus likely a consequence of different rates of EVET due to the shifted vibrational energies of the deuterated solvents. It may be counterintuitive that EVET-mediated PL quenching occurs at all for NCs with a silica shell, which spatially isolates the luminescent core from the surrounding medium. However, silica shells grown *via* microemulsion methods are typically (micro-)porous,^9,27,36,37^ so that small molecules or ions can penetrate and reach sites close to the luminescent core where they are efficient acceptors for EVET.

Fig. 1e–g show the energies of some relevant transitions in Er^3+^ that can be bridged by EVET (see also the ESI;† section S2), and how they match the vibrational absorption spectrum of the various normal and deuterated solvents used. Different solvents clearly offer different resonances between vibrational modes and Er^3+^ transitions, and the resulting EVET-mediated quenching affects the spectral distribution of the upconversion PL (Fig. 1d) among the red and green emissions. For example, the green contribution of the PL is low in OH-containing solvents H_2_O and ethanol compared to their deuterated analogs. The underlying mechanism could be twofold: EVET from the green-emitting levels (^4^S_3/2_ and ^2^H_11/2_) to OH-vibrations quenches the green PL directly, or EVET from the intermediate near-infrared-emitting ^4^I_11/2_ level could quench the green PL indirectly. The latter effect establishes a population of Er^3+^ ions in the ^4^I_13/2_ state from which energy-transfer upconversion to the red-emitting ^4^F_9/2_ level occurs. As upconversion is a complex higher-order process, pinpointing the exact contributions of the different EVET donor transitions (Fig. 1f) and vibrational acceptors (Fig. 1g) to the observed changes in the PL spectrum is challenging. To identify the dominant EVET-mediated quenching pathways, it is important to compare the excited-state dynamics of different energy levels when excited resonantly.

**Fig. 2:**
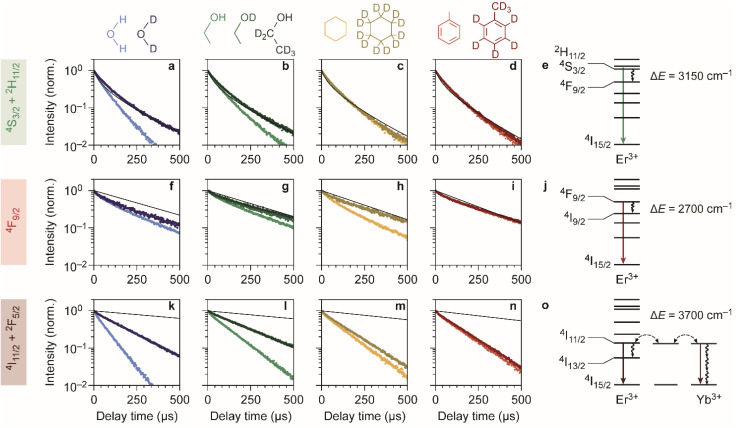
Quenching of Er^3+^ levels in different chemical environments. (a–d) Photoluminescence decay curves of the green-emitting levels in water, ethanol, cyclohexane, and toluene are shown in light shades of blue, green, yellow and red. Decay curves measured in deuterated isotopologues are shown in darker shades of the corresponding colors. Theoretical predictions for decay in the absence of EVET are shown as black lines. (e) Schematic of EVET quenching of the green-emitting donor level. (f–j) Same as a–e, but for the red-emitting energy levels. (k–o) Same as a–e, but for the near-infrared-emitting energy levels.

Fig. 2a–d shows the experimentally obtained decay curves of the green-emitting levels of our Er^3+^–Yb^3+^ co-doped NCs upon resonant excitation. We used the silica-coated NCs to measure the decay dynamics in the polar solvents water and ethanol, and the oleic-acid-coated NCs for measurements in cyclohexane and toluene. For comparison, we also show theoretical predictions of the decay dynamics in the absence of EVET-mediated quenching (ESI,† section S3), based on the NC-cavity model for radiative decay rates^40^ and a shell model for ion-to-ion energy transfer that predicts the rate of cross relaxation.^11,41–43^ Almost all experimental decay curves of the green-emitting levels closely follow the decay dynamics predicted without EVET, including the measurements with NCs dispersed in D_2_O, ethanol-d1, and ethanol-d6, but not in H_2_O and ethanol (Fig. 2a–d). This implies that EVET is efficient to acceptors with OH-groups but not to their deuterated counterparts, which have a lower vibrational energy. This is consistent with the observation that the ^4^S_3/2_ → ^4^F_9/2_ donor transition (3000–3400 cm^−1^), which quenches the green PL, has good overlap with the absorption band of O–H stretching vibrations (3100–3700 cm^−1^) but not with the deuterated analog (2300–2700 cm^−1^; Fig. 1f–g).

For the red-emitting ^4^F_9/2_ level, cross-relaxation plays no significant role because energy transfer to neighbouring erbium ions in the ^4^I_15/2_ ground-state level is prevented by a large energetic mismatch. The theoretical decay curves based on radiative decay closely follow most experimentally obtained decay dynamics (Fig. 2f–i). However, faster decay rates are exhibited by NCs dispersed in H_2_O, D_2_O, ethanol, and in particular cyclohexane. This may be explained in terms of spectral overlap of the ^4^F_9/2_ → ^4^I_9/2_ transition (2600–3000 cm^−1^; Fig. 1f), which quenches the red PL, and the vibrational energies of the medium around the NCs. Aliphatic C–H stretching vibrations of cyclohexane (2800–3000 cm^−1^) are resonant with the quenching transition and therefore strong EVET acceptors, while C–D vibrations (2100–2300 cm^−1^) and aromatic C–H vibrations (3000–3100 cm^−1^) are not.^11^ This stresses the importance of resonance as a criterion for EVET to occur. Significant EVET from the ^4^F_9/2_ → ^4^I_9/2_ transition to O–H stretching vibrations is not expected *a priori*, as the (broad) infrared absorption band measured in liquid phase lies at higher energies (Fig. 1g). However, O–H and O–D stretching vibrations inside or around the silica matrix have mode energies shifted to ∼2750–3500 cm^−1^ and ∼2000–2700 cm^−1^, respectively, as we will demonstrate later (see Fig. 4). This could facilitate EVET-mediated quenching of the red-emitting level by both O–H and O–D vibrations depending on the molecule. For now, we conclude that the ^4^F_9/2_ → ^4^I_9/2_ EVET donor transition can couple to acceptors with aliphatic C–H vibrations, O–H vibrations, and O–D stretching vibrations.

Our upconversion NCs also emit in the near-infrared PL, but the origin is complex due to energy migration between the Yb^3+ 2^F_5/2_ and Er^3+ 4^I_11/2_ levels, which both emit around 1000 nm and both contribute to the decay curve. Surprisingly, all decay curves of the near-infrared-emitting levels show dynamics that are substantially faster than the radiative decay (Fig. 2k–n). The strongest quenching is observed for NCs dispersed in H_2_O and ethanol, which is consistent with the spectral overlap between the ^4^I_11/2_ → ^4^I_13/2_ donor transition of Er^3+^ (at 3500–3800 cm^−1^) and the O–H stretching vibration (see Fig. 1f–g). An important contribution may however be EVET from the ^2^F_5/2_ → ^2^F_7/2_ donor transition of Yb^3+^ (at ∼10 200 cm^−1^) to combination-vibrations (2*ν*_1_ + *ν*_3_; ref. 44) of hydroxyl groups, as proposed previously by other groups.^9,45^ The EVET rate from the near-infrared-emitting levels is effectively enhanced by fast energy migration among and between Er^3+^ and Yb^3+^ ions, facilitating transfer of energy to the NC surface followed by quenching. In contrast, energy migration of green- and red-emitting levels can only proceed *via* the sparse sublattice of Er^3+^ dopants, which explains why its EVET-mediated quenching of visible emissions is much weaker. Strong quenching by OH vibrations is also understandable in view of the high oscillator strength of the O–H stretch vibration.

Another aspect that influences the efficiency of EVET is the density of acceptor vibrations in the NC's immediate environment. At higher acceptor densities, donors can transfer their energy to different acceptors, which favors EVET over radiative decay. This consideration explains the difference between strong near-infrared-quenching by water and weaker quenching by ethanol (Fig. 2k and l): water has a higher density of OH-vibrations than ethanol in the bulk liquid and may furthermore penetrate the silica shell more effectively. Similarly, the relatively weak quenching observed for silica-coated NCs in deuterated ethanol-d1 and ethanol-d6 (Fig. 2l) compared to oleic-acid coated NCs in toluene and cyclohexane (Fig. 2m and n) may be attributed to different local densities of CH-vibrations, which are affected by limited penetration of ethanol into silica shell. Note that this density refers to the number of vibrations per unit volume, rather than the number of molecules.

Using the insights into the PL decay dynamics, we can now explain the observed trends of the upconversion PL spectrum in different solvents (Fig. 1d). Hydroxyl groups directly quench the green-emitting levels. In addition, they quench the near-infrared-emitting ^4^I_11/2_ level of Er^3+^, transferring population to the ^4^I_13/2_ level from which energy-transfer upconversion populates the red-emitting ^4^F_9/2_ level. Hence, quenching by OH groups changes the upconversion pathways through which the red-emitting ^4^F_9/2_ level is populated, but does not necessarily reduce the ^4^F_9/2_ population. Both effects result in a lower relative contribution of green upconversion PL for NCs in media with OH groups (*e.g.*, normal water and ethanol). Another important observation is the predominant green upconversion PL in normal cyclohexane compared to the lower green contribution in other organic solvents. This difference can be attributed to the aliphatic C–H EVET acceptor, which strongly quenches the red-emitting level.

Although we can explain many trends in the PL spectrum and lifetimes (Fig. 1 and 2) in terms of EVET involving a single fundamental molecular vibration that acts as an acceptor, quenching of the near-infrared-emitting ^4^I_11/2_ level of Er^3+^ by deuterated water and ethanol, or in both isotopologues of cyclohexane and toluene (Fig. 2k–n) remains complex. Native high-energy C–H or O–H vibrations of oleic acid or silica (silanol groups), or native high-energy lattice vibrations,^46,47^ which we ignored in our discussion so far, may play a significant role as EVET acceptors. Alternatively, complex EVET mechanisms involving combination-vibrations of the acceptor may be responsible for quenching in these environments.^9,45^ Beyond Er^3+^, experiments on NaYF_4_:Ho^3+^ NCs (ESI;† section S4) show a complex interplay between EVET and intrinsic decay dynamics, *i.e.*, radiative decay and cross-relaxation. It is thus difficult to predict *a priori* which transitions will experience substantial EVET-mediated quenching.

### Photoluminescence quenching by molecules from a vapor phase

**Fig. 3:**
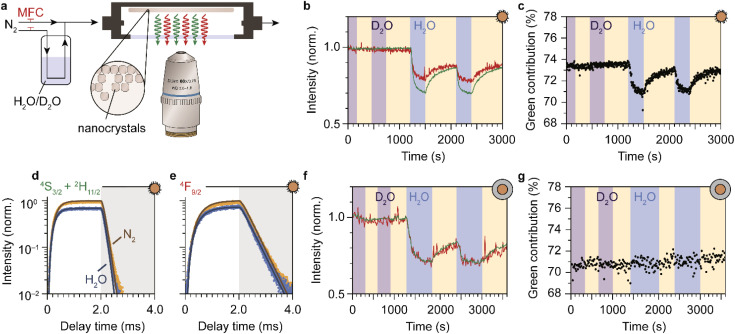
Detection of water vapor with lanthanide-doped upconversion NCs. (a) A drop-casted layer of upconversion NCs is exposed to a constant flow of dry nitrogen carrier gas with controllable amounts of water vapor, using mass-flow controllers (MFCs). The sample is excited in the infrared and visible photoluminescence is collected using a microscope objective. (b) Normalized green and red photoluminescence intensities of oleic-acid-coated NaYF_4_:Er^3+^(2%),Yb^3+^(18%) NCs, measured under dry nitrogen flow (yellow-shaded areas) or in the presence of water vapor at a relative humidity *p*/*p*_0_ = 0.17. Blue and purple-shaded areas correspond to a flow of H_2_O and D_2_O, respectively. (c) Same as b, but for the green contribution to the upconversion PL. (d and e) Traces of the green and red photoluminescence excited at 980 nm under dry nitrogen flow (yellow) and in the presence of water vapor (blue). The laser was operated in block-pulsed mode, where it was on for the first 2 ms (unshaded area) and off for the last 2 ms (shaded area). Solid lines are fits to the rate-equation model (ESI;† section S7) (f and g) Same as b–c, but for SiO_2_-coated NCs. The higher noise level (weaker emission) may be the result of a thinner NC layer.

So far, we have demonstrated that the PL from lanthanide dopants is affected by the solvent in which NCs are dispersed. Now we extend our study by investigating if PL quenching also occurs for EVET acceptors in a gas phase. We measured the upconversion PL of a layer of oleic-acid-coated NaYF_4_:Er^3+^(2%),Yb^3+^(18%) NCs, exposed to a flow of nitrogen gas with controllable amount of water vapor (see Fig. 3a) and excited at excitation powers of approximately 10 W cm^−2^. At these powers, the green PL contributes between 70 and 74% of the total visible upconversion PL, depending on the gas flow (ESI,† section S5–6). Fig. 3b shows the changes in the green and red PL intensities of the NCs during a prolonged experiment in which we switch between dry nitrogen flow (yellow shaded areas) and flows containing D_2_O (purple) or H_2_O vapor (blue). The upconversion PL intensity and spectrum of the NCs are unaffected by D_2_O vapor. However, introducing H_2_O causes a rapid drop of both the red and green upconversion PL, by 20–30%. The PL recovers to ∼90% of the initial intensity within 10 minutes upon switching back to dry nitrogen. After 10 min the PL intensity has not yet reached a steady state, implying that further recovery is possible but would require more time (not tested experimentally). Besides a drop in intensity, the relative contributions of green and red upconversion PL change by approximately 4–5% upon introducing water vapor to the gas flow (Fig. 3c). This effect is approximately 10 times weaker than the difference of 40% observed between the experiments with NCs dispersed in liquid H_2_O and D_2_O (Fig. 1d). The amplitude of the decay curves of the green and red PL drops by 31 and 26%, while the apparent decay rates remain approximately unchanged (see Fig. 3d and e).

We ascribe the drop of the upconversion PL intensity to EVET dominated by quenching of the intermediate near-infrared-emitting ^4^I_11/2_ level. This is consistent with a simple rate-equation model (ESI,† section S7) that describes upconversion as a three-level system (near-infrared, red and green), ignoring the effects of cross-relaxation or energy migration. The model predicts that the steady-state amplitude of the decay curve depends quadratically on the decay rate of the near-infrared-emitting level, and linearly on the red- and green-emitting levels. In this framework, quenching of the Yb^3+ 2^F_5/2_ and the Er^3+ 4^I_11/2_ near-infrared-emitting levels by ∼10% causes the 20–30% quenching of visible upconversion PL.

Although quenching of the near-infrared-emitting level by water adsorbates is approximately 10 times weaker than quenching by liquid water (Fig. 2k), the effect is much stronger than expected based on the density of molecules, which differs by 4–5 orders of magnitude. Assuming that the rate of EVET scales linearly with the local density of OH-acceptors, this implies that water molecules from the gas flow accumulate in a small volume around the NC—*i.e.*, significant room-temperature adsorption of water molecules on the NC surface causes the observed EVET-mediated quenching of upconversion PL. The (partial) recovery of the PL upon purging with dry nitrogen implies that room-temperature desorption of adsorbates also occurs readily.

The PL from the silica-coated NCs is also quenched upon exposure to a gas flow containing water vapor and recovers partially upon switching back to dry nitrogen flow (Fig. 3f, g and ESI,† section S7). The time scale of PL recovery is, however, slower than observed for the oleic-acid-coated NCs. This can be attributed to hampered desorption of water molecules due to stabilization of adsorbates in the microporous silica structure. Moreover, the silica-coated NCs exhibit constant relative contributions of the green and red upconversion PL, in contrast to the oleic-acid-coated NCs (compare Fig. 3c and g). The difference may be a result of different “background quenching” induced by the oleic acid ligands and the silica matrix, which may influence the contributions of different upconversion pathways and the relative importance of any potential water-induced effects. This is reflected by the green contribution to the total upconversion PL, which is higher for oleic-acid-coated NCs (73–74% under dry nitrogen flow) compared to silica-coated NCs (70–71%).

**Fig. 4:**
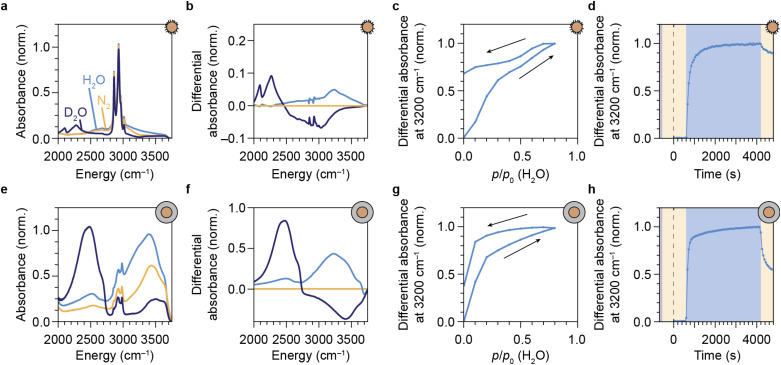
Monitoring the adsorption of water vapor with ATR-FTIR spectroscopy. (a) Infrared absorption spectra of a layer of oleic-acid-coated NaYF_4_:Er^3+^(2%),Yb^3+^(18%) NCs, under dry N_2_ flow (yellow), and under flows of *p*/*p*_0_ = 0.20 H_2_O (blue) and D_2_O (purple). (b) Differential infrared absorption spectra, relative to the absorption under dry N_2_ flow. (c) Differential absorbance at 3200 cm^−1^ as a function of the relative water pressure *p*/*p*_0_ (H_2_O). Arrows indicate the adsorption and desorption isotherms. (d) Normalized differential absorbance at 3200 cm^−1^, measured under dry nitrogen flow (yellow-shaded areas) or in the presence of water vapor (*p*/*p*_0_ = 0.17; blue-shaded area). The sample was purged with D_2_O vapor before starting the experiment. (e–h) Same as a–d, but for SiO_2_-coated NCs.

We have shown that our NCs respond to water vapor in the atmosphere by a reversible spectral response in the form of a change in intensity and the relative contribution of green to the upconversion PL. This implies that our NCs can be used and re-used to probe vibrations of gaseous EVET acceptors that adsorb and desorb to and from the surface. For a direct proof of water adsorption/desorption, we performed attenuated total reflection Fourier-transform infrared spectroscopy (ATR-FTIR) on films of NCs under the applied gas flows. The infrared absorption spectrum of a layer of oleic-acid-coated NCs features a strong signal around 2900 cm^−1^ due to C–H vibrations of the oleic acid capping ligands (Fig. 4a). Introducing (deuterated) water vapor to the flow induces an increasing absorption band corresponding to the O–H (O–D) stretching vibration around 2750–3600 cm^−1^ (2000–2700 cm^−1^), as is clear from the differential absorption spectrum (Fig. 4b). Increasing the concentration of water vapor leads to an upcoming differential absorption signal of the O–H band (Fig. 4c), confirming that water adsorbs onto the surface of the NCs (see also the ESI,† section S10). The approximately linear adsorption isotherm (ESI, Fig. S9†) is characteristic for nonpolar surfaces with weak water–surface interactions (type III or V according to the IUPAC classification^48^) and implies that water adsorbates cluster around a few favorable adsorption sites rather than forming a monolayer. The absorbance decreases again upon lowering the water concentration in the flow, but adsorption/desorption of H_2_O is not fully reversible. The same behavior is observed for D_2_O adsorption/desorption (see ESI,† section S8). Fig. 4d shows the dynamics of H_2_O adsorption and desorption: upon introducing water vapor, the absorbance signal quickly increases on the time scale of minutes. Removing water vapor from the flow again results in a drop of the signal on time scales of ∼10 min, but recovery is far from complete. This indicates that a large fraction of water molecules remains in the film of NCs. While the behavior observed in Fig. 4d is qualitatively similar to that in Fig. 3b, the near-complete recovery of the upconversion PL seems inconsistent with the far-from-complete recovery of the infrared absorbance. Hampered desorption from the film of the oleic-acid-coated NCs may be explained in terms of strong interaction of water molecules with oleic acid ligands, possibly forming structures in which water is stabilized.^49–51^ Such a strong interaction is implied by the shift of C–H stretching absorption lines in the 2800–3000 cm^−1^ range (Fig. 4b). It is therefore possible that remaining water molecules detected by ATR-FTIR spectroscopy reside in oleic-acid-stabilized structures and are not localized at the NC surface, rendering them inefficient EVET acceptors that cause only minor PL quenching.

The silica-coated NCs exhibit an absorption band in the 2750–3500 cm^−1^ range due to native silanol Si–O–H vibrations (Fig. 4e). The absorption feature around 2900–3000 cm^−1^ originates from residual surfactants (IGEPAL CO-520) used in the microemulsion method of silica shell growth.^24,27^ An absorption band of condensed O–H vibrations appears when flowing water vapor over the layer of NCs (see Fig. 4f). The isotherm obtained from the O–H stretching band as a function of applied humidity (Fig. 4g and ESI, Fig. S9†) is qualitatively different from the isotherm measured for the hydrophobic oleic-acid-coated NCs (Fig. 4c). The most important difference is that the silica shell facilitates significant adsorption of water already at a low relative humidity, typical for hydrophilic microporous materials (compare Fig. 4c, g and ESI, Fig. S9a, b†). This results in a type I-b adsorption isotherm according to the IUPAC classification.^48^ The rapid initial adsorption of water at low pressure corresponds to the filling of the micropores of the hydrophilic porous network of the silica shell with water. Desorption of water adsorbates is incomplete, but more substantial than for the oleic-acid-coated NCs (compare Fig. 4h with Fig. 4d). This more substantial release of water adsorbates, however, does not lead to a larger recovery of the upconversion PL (compare Fig. 3c and g). We propose that, despite desorption of most water molecules from the silica shell, a significant fraction of water adsorbates remains close to the surface of the luminescent cores, possibly kinetically stabilized by slow diffusion through the silica pores. These remaining adsorbates likely reside deep within the silica shell—close to the luminescent NC core—and are thus important quenchers. Instead of purging with dry nitrogen gas, a faster method to remove these quenchers for recovery of the upconversion PL is to exchange O–H groups with O–D groups, which happens rapidly upon flushing with D_2_O vapor (Fig. 4f, see ESI,† section S9).

Optical spectroscopy (Fig. 3) and ATR-FTIR spectroscopy (Fig. 4) are thus both capable of tracking adsorption of molecules on the surface of lanthanide-doped NCs. However, while the PL of the NCs is affected predominantly by short-range (up to several nm) EVET-mediated quenching, ATR-IR spectroscopy also probes vibrations of molecules adsorbed in a larger volume, including interparticle voids. When combined, these two complementary techniques provide information about quenching induced by surface adsorbates.

## Discussion

Our experiments are a proof of concept, demonstrating that sensing of molecular vibrations based on EVET-mediated quenching of the PL from lanthanide-doped NCs is possible both in the liquid and gas phase. Important parameters that govern the efficiency of EVET are (a) the match of the energy gap between the emitting levels of the lanthanide ion and the vibrational energy of the EVET acceptor, (b) the oscillator strengths of the electronic donor transition of the lanthanide ion and of the vibrational mode that acts as an acceptor, and (c) the density of EVET acceptors in the volume that extends several nanometers from the NC surface. While we can describe some EVET-mediated quenching processes in terms of these three considerations, (the degree of) quenching by other EVET acceptors remains difficult to understand. A reason for this might be that combinations of different vibrations may act as EVET acceptors and thereby quench lanthanide levels, as we proposed for quenching of the near-infrared-emitting level of Er^3+^.

Despite these difficulties, we envision that detection and identification of different molecules with a variety of functional groups may be possible using NCs doped with different lanthanide ions. There is much room for exploration when taking advantage of the wide choice of available lanthanide ions, which all feature different energy separations that could match the vibrational energy of various molecules with different functional groups. For example, Eu^3+^ may be a promising candidate for detection of carbonyl moieties as the ^5^D_1_ → ^5^D_0_ relaxation transition may couple to the C=O vibration with a mode energy of 1700–1800 cm^−1^. Another example is Ho^3+^, where EVET from the ^5^F_3_ → ^5^S_2_ transition to C–D vibrations (∼2000 cm^−1^) may alter the shape of the PL spectrum. The sensitivity of lanthanide-doped NCs for the detection of molecules can likely be enhanced by using new oleate-based synthesis procedures that avoid the incorporation of hydroxide ions in the NC host lattice.^52^

We foresee that a molecule-specific response may become possible when exploiting the rich surface chemistry and tunable microporosity^53–55^ of a (functionalized) silica shell that acts as a molecular sieve with adjustable sieving and adsorption properties. This would improve the selectivity of EVET-based sensing. Molecule-specific sensing is otherwise challenging, as different molecules have the same functional groups (compare H_2_O and ethanol; Fig. 2) and the spectrally broad lanthanide transitions often overlap with multiple molecular vibrations (compare Fig. 1f and g). Alternatively, combining different lanthanide probes with partially overlapping transitions could improve the molecule-specific response.

Our experiments also shed light on the ongoing debate about the peculiar observation of reversible thermal enhancement of upconversion PL in lanthanide-doped NCs.^56–58^ While complex quenching mechanisms have been proposed,^56,59,60^ our results are consistent with the simple explanation that thermal enhancement stems from temperature-induced removal of water adsorbates that act as EVET acceptors.^16,61–63^

## Conclusions

We have shown that the PL of lanthanide-doped (upconversion) NCs is sensitive to short-range EVET-mediated quenching by nearby molecular vibrations. The energy level-structure of lanthanide ions contributes to a unique PL spectrum, which is altered when different energy levels are quenched at different rates. We have demonstrated this effect for NCs in solution by measuring the PL decay dynamics in isotopologues of water, ethanol, cyclohexane, and toluene. The comparison of isotopologues highlights the sensitivity of the lanthanide PL to the vibrational spectrum of the surrounding solvents. In many cases, the quenching strength can be rationalized based on the energetic (mis)match of the lanthanide EVET donor and the EVET acceptor mode. We have shown that efficient quenching of the PL does not necessarily require the NCs to be completely immersed by a medium of quenching molecules. Instead, quenching can already be induced by EVET to molecules adsorbed at the NC surface, such as water. Using the concept of EVET, lanthanide-doped NCs have potential as nanosensors, detecting vibrational modes in their local environment by giving a response in the form of a change in the PL spectrum and lifetimes.

## Author contributions

The manuscript was written through contributions of all authors. All authors have given approval to the final version of the manuscript.

## Conflicts of interest

There are no conflicts to declare.

## Supplementary Material

NR-015-D3NR02997B-s001
