# Supplementary material for: Probing nearby molecular vibrations with lanthanide-doped nanocrystals
Source: Nanoscale. 2023 Oct 3;15(41):16601–11. doi: 10.1039/d3nr02997b (PMC10600830; doi:10.1039/d3nr02997b)
Supplement: NR-015-D3NR02997B-s001 [file NR-015-D3NR02997B-s001.pdf]

Electronic Supplementary Material (ESI) for Nanoscale.
This journal is © The Royal Society of Chemistry 2023

Supporting Information for

Probing nearby molecular vibrations with lanthanide-doped nanocrystals

*Mark J. J. Mangnus[1,2], Vincent R. M. Benning[1,2], Bettina Baumgartner[1], P. Tim Prins[1], Thomas P. van Swieten[1,3], Ayla J. H. Dekker[1,2], Alfons van Blaaderen[2], Bert M. Weckhuysen[1], Andries Meijerink[3], and Freddy T. Rabouw[1,2]**

[1] Inorganic Chemistry and Catalysis group, Debye Institute for Nanomaterials Science and Institute for Sustainable and Circular Chemistry, Utrecht University, Universiteitsweg 99, 3584 CG Utrecht, The Netherlands

[2] Soft Condensed Matter group, Debye Institute for Nanomaterials Science, Utrecht University, Princetonplein 1, 3584 CC Utrecht, The Netherlands

[3] Condensed Matter and Interfaces group, Debye Institute for Nanomaterials Science, Utrecht University, Princetonplein 1, 3584 CC Utrecht, The Netherlands

* Corresponding author. E-mail: f.t.rabouw@uu.nl

Section S1: Upconversion emission spectra in different solvents

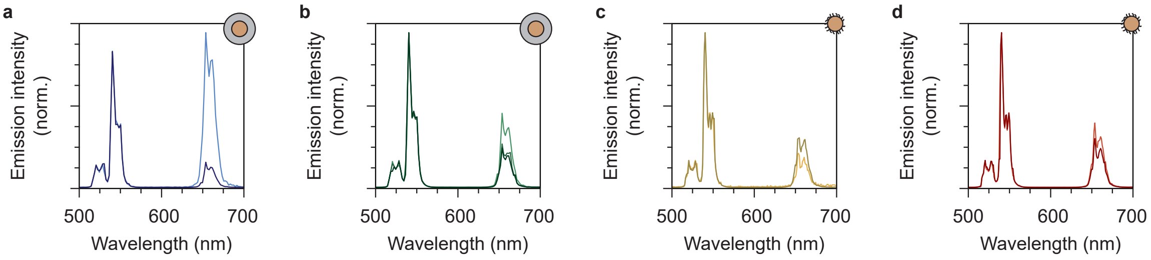

Figure S1 | Upconversion emission spectra in different solvents. **(a)** Upconversion emission spectra of silica-coated $NaYF_4$:Er^{3+}(2%),Yb^{3+}(18%) NCs dispersed in normal water and deuterated water (light and dark blue spectra, respectively). **(b)** Same as **a**, but for silica-coated NCs dispersed normal and deuterated isotopologues of ethanol. **(c)** Same as **a**, but for oleic-acid-coated $NaYF_4$:Er^{3+}(2%),Yb^{3+}(18%) NCs dispersed in normal and deuterated cyclohexane. **(d)** Same as **c**, but for oleic-acid-coated NCs dispersed in normal and deuterated toluene.

Section S2: Oscillator strengths of electronic relaxations in lanthanides

Following Ref. S1, the oscillator strength of an electric dipole transition from an initial electronic state with quantum numbers S, L and J, $\langle 4f, SLJ\rangle$, to a final state with quantum numbers S', L' and J', $\langle 4f, S'L'J'\rangle$, can be calculated using Judd–Ofelt theory:

$$f_{\mathrm{ED}} = C\frac{\xi^*}{2J+1}\frac{\chi}{n}\sum_{\lambda=2,4,6}\Omega_\lambda |\,\langle 4f, SLJ|U^{(\lambda)}|4f, S'L'J'\rangle\,|^2, \tag{1}$$

where C is a prefactor ($1.1\times10^{11}\mathrm{cm}^{-1}$), ξ^* is the transition barycenter, χ is the local-field factor (see Section S3) and n is the refractive index of the medium. Ω_λ are the three lattice-specific Judd–Ofelt parameters (4.97×10^{-20}, 1.16×10^{-20}, and 2.03×10^{-20} cm^{-2} for Ω_2, Ω_2 and Ω_2 in β-$NaYF_4$; Ref. S1) and $U^{(\lambda)}$ are the electric dipole tensor operators. The matrix elements $|\,\langle 4f, SLJ|U^{(\lambda)}|4f, S'L'J'\rangle\,|^2$ are tabulated in Ref. S2.

We used the absorption spectrum of bulk β-$NaGdF_4$ powder (Figure S2a; Obtained from Ref. Freddy) to estimate the transition energies and widths. First, we determined the energies of the different levels ν^* (see Figure S2b–c) and (ignoring crystal-field splitting of the $^4I_{15/2}$ ground state of erbium) approximated spectra of the oscillator strength T of a transition from initial state i to final state j as

$$T_{i,j}(\xi) = \int A(\nu_i^* - \xi)\, A(\nu_j^*)\, \mathrm{d}\xi, \tag{2}$$

where ξ is the energy difference from the transition barycenter. Theoretical spectra in Figure 1f (main text) are based on the shape $T_{i,j}(\xi)$, combined with the calculated values of f_{ED} (see Table S1) as an amplitude.

Initial state	Final state	$U^{(2)}$	$U^{(4)}$	$U^{(6)}$	ξ^* (cm^{-1})	f_{ED}	σ (cm^{-1})
$^2H_{11/2}$ + $^4S_{3/2}$	$^4F_{9/2}$	0.3790	0.0236	0.0008	3191	6.1×10^{-8}	65
$^4F_{9/2}$	$^4I_{9/2}$	0.1120	0.0061	0.0203	2815	2.8×10^{-7}	75
$^4I_{11/2}$	$^4I_{13/2}$	0.0332	0.1706	1.0915	3701	1.4×10^{-6}	47

Table S1 | Relaxation transitions of the erbium excited state. Matrix elements of $U^{(\lambda)}$, obtained from Ref. S2. Transition oscillator strengths are calculated based on Eq. 1. Transition barycenters ξ^* and widths σ are determined based on the absorption spectrum of bulk β-$NaGdF_4$ powder (Figure S2).

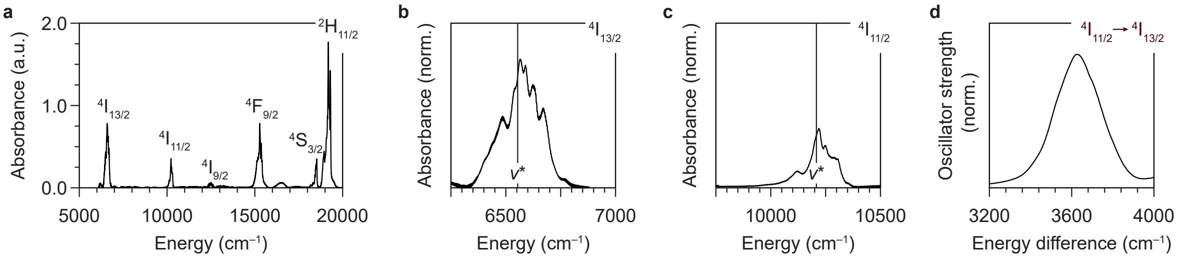

Figure S2 | Determining spectra of transition oscillator strengths. **(a)** Absorption spectrum of bulk β-$NaGdF_4$:Er^{3+}(1%) powder. Transitions from the $^4I_{15/2}$ ground state to several excited states are labeled. **(b)** Zoom-in of **a** around the $^4I_{15/2}\rightarrow{}^4I_{13/2}$ absorption transition. ν^* indicates the transition center of gravity. Same as **b**, but for $^4I_{15/2}\rightarrow{}^4I_{11/2}$ absorption transition. **(d)** Spectrum of the oscillator strength of the $^4I_{11/2}$ $\rightarrow$ $^4I_{13/2}$ relaxation.

Section S3: Intrinsic decay dynamics of nanocrystals

For lanthanide dopants in NCs with dimensions smaller than the wavelength of light, the radiative decay rate differs depending on the photonic environment according to the NC-cavity model:[S3]

$$\Gamma_{\mathrm{rad}}(n) = \Gamma_0 n \chi^2. \tag{3}$$

Here, Γ_0 is the decay rate of the emitter in vacuum, n is the solvent refractive index, and χ is the local-field factor, which accounts for the fact that the local electric field amplitude of photon states at the position of the emitter is different from that of the macroscopic electric field. For spherical NCs with refractive index n_{NC}, there is an analytical expression for χ^2:[S3]

$$\chi^2_{\mathrm{sph}} = \left(\frac{3n^2}{2n^2 + n^2_{\mathrm{NC}}} \right)^2. \tag{4}$$

In Figure 2 (main text), we used the NC-cavity model (Eq. 4) and literature values of the radiative decay rates (Ref. S4) to calculate theoretical decay curves of NCs dispersed in different solvents, where the only decay pathway is radiative.

For the green-emitting levels of erbium, cross-relaxation plays an important role in the decay dynamics.[S4] The rate of cross-relaxation from a central donor ion depends on its local environment in terms of the number of acceptor ions and the corresponding donor–acceptor distances (with an r^{-6}-dependence). Due to random occupation of rare-earth sites by lanthanide ions, an ensemble of Er-doped NCs features a distribution of cross-relaxation rates. Importantly, donor–acceptor distances are discrete and follow a shell-like structure with nearest neighbors, next-nearest neighbors, etc.[S4–S7] The β-$NaYF_4$ unit cell has two different rare-earth sites: the A-site that is 100% available for lanthanide ions, and the B-sites that is 50% occupied by Na ions.[S8] For a statistical distribution of lanthanide ions, there is an analytical expression for the (multi-exponential) contribution of cross-relaxation $X(t)$ to the total decay dynamics:[S4]

$$\begin{aligned} X(t) =& \frac{2}{3} \left\{ \prod_i \left(1 - \phi + \phi \mathrm{e}^{-C_{\mathrm{X}} t / r_i^6} \right)^{n_i} \times \left(1 - \frac{\phi}{2} + \frac{\phi}{2} \mathrm{e}^{-C_{\mathrm{X}} t / r_i^{*6}} \right)^{n_i^*} \right\} \\ &+ \frac{1}{3} \left\{ \prod_i \left(1 - \frac{\phi}{2} + \frac{\phi}{2} \mathrm{e}^{-C_{\mathrm{X}} t / r_i^6} \right)^{n_i} \times \left(1 - \phi + \phi \mathrm{e}^{-C_{\mathrm{X}} t / r_i^{*6}} \right)^{n_i^*} \right\}. \end{aligned} \tag{5}$$

The dynamics of cross-relaxation has contributions of erbium donors in A and B sites, with acceptors positioned in shells i, with n_i rare-earth sites at distances r_i and r_i^*. Cross-relaxation becomes faster at higher doping concentrations ϕ. Theoretical decay curves in Figure 2a–d (main text) contain contributions of radiative decay (Eqs. 3–4), Er–Er cross-relaxation and Er–Yb cross-relaxation (Eq. 5). We used values $C_{\mathrm{Er,Er}}$ and $C_{\mathrm{Er,Yb}}$ from Ref. S4. Note that the shell model for cross-relaxation does not account for energy migration among lanthanide ions and neglects finite-size effects. The theoretical curves in Figure 2a–d therefore serve as a rough estimation of the intrinsic decay dynamics of the green-emitting levels.

Section S4: EVET-mediated quenching in Ho^{3+}-doped NCs

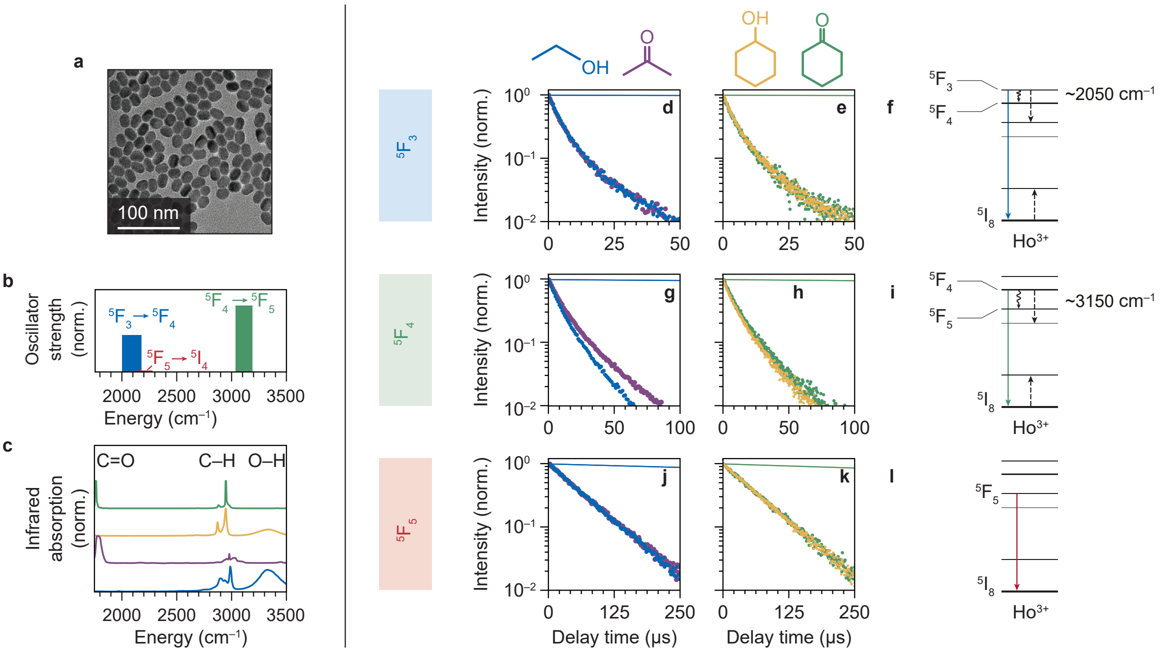

Figure S3 | Quenching of holmium levels in different chemical environments. **(a)** Transmission electron microscopy images of the oleic-acid-coated $NaYF_4$:Ho^{3+}(5%) NCs, with a diameter of 31 ± 3 nm. A ligand-exchange procedure yielding BF_4^--capped NCs, described in Ref. S9, was performed prior to dispersing the NCs in polar solvents. **(b)** Normalized oscillator strengths and energies of important relaxation transitions in Ho^{3+}, calculated using the procedure described in Section S2.[S2] Oscillator strengths f_{ED} of relaxations from the blue-, green- and red-emitting levels are 3.4×10^{-7}, 6.2×10^{-7} and 4.9×10^{-9}. **(c)** Infrared absorption spectra of solvents molecules, reproduced from Ref. S10. **(d–e)** PL decay curves of the blue-emitting levels in acetone, ethanol, cyclohexanol and cyclohexanone are shown in purple, blue, green and yellow. Theoretical predictions for radiative decay in the absence of EVET and cross-relaxation are shown as solid lines.[S11] **(f)** Schematic of EVET quenching of the blue-emitting donor level. Cross-relaxation is indicated by dashed arrows. **(g–i)** Same as **d–f**, but for the green-emitting level. **(j–l)** Same as **d–f**, but for the red-emitting level. The blue, green and red decay curves were obtained by exciting the sample at 447, 535 and 638 nm, and collecting the luminescence at 487, 542 and 645 nm. The setup used was the same as described in the Methods section.

Section S5: Dependence of upconversion luminescence on excitation power and profile

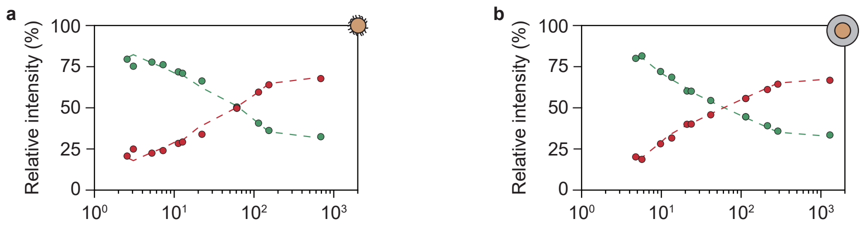

Figure S4 | Power-dependence of the upconversion emission spectrum. **(a)** Relative contributions of the green and red upconversion emissions from a film of oleic-acid-coated NaYF$_4$:Er^{3+}(2%),Yb^{3+}(18%), as a function of power density. The green and red datapoints correspond to measurements under dry nitrogen flow, and the dashed lines correspond to the same measurements under a flow containing H$_2$O vapor at $p/p_0 = 0.17$. Note that the power dependence is approximately the same under these two different gas flows. **(b)** Same as **a**, but for a film of silica-coated NaYF$_4$:Er^{3+}(2%),Yb^{3+}(18%) NCs.

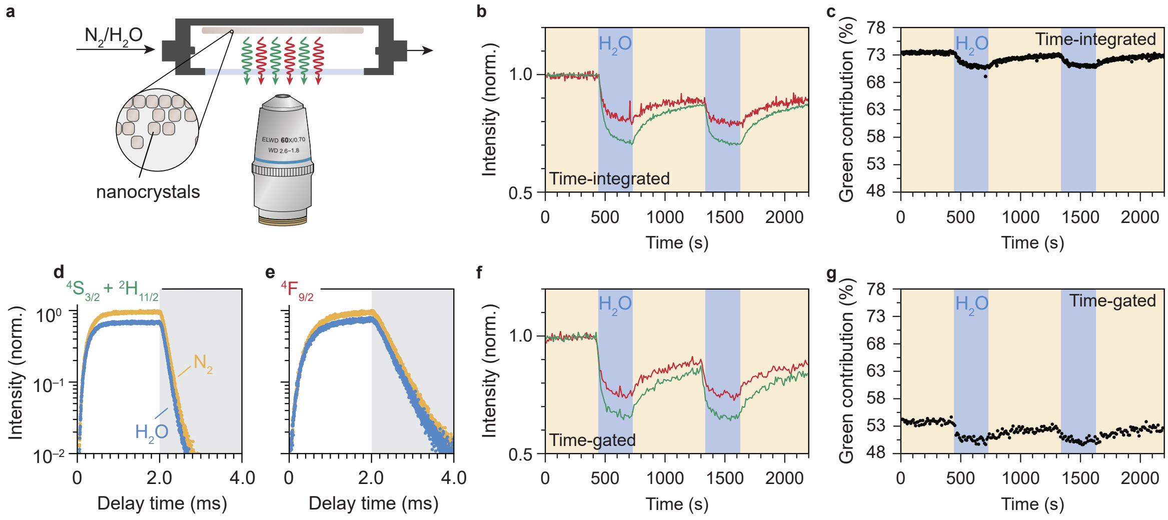

Figure S5 | Effect of time gating on the detection of water vapor. **(a)** A drop-casted layer of upconversion NCs is exposed to a constant flow of dry nitrogen carrier gas with controllable amounts of water vapor. **(b)** Normalized green and red emission intensities of oleic-acid-coated NaYF$_4$:Er^{3+}(2%),Yb^{3+}(18%) NCs, measured under dry nitrogen flow (brown-shaded areas) or in the presence of water vapor ($p/p_0 = 0.17$; blue-shaded areas). **(c)** Same as **b**, but for the contribution of green emission to the upconversion PL. **(d–e)** PL decay curves of the green and red emissions excited at 980 nm under dry nitrogen flow and in the presence of water vapor. The laser was operated in block-pulsed mode, where it was on for the first 2 ms (unshaded area) and off for the last 2 ms (shaded area). **(f–g)** Same as **b–c**, but using only the photons emitted in the second half of the block pulses (at times when the laser was off; grey areas in **d–e**).

Section S6: Emission spectra under different gas flows

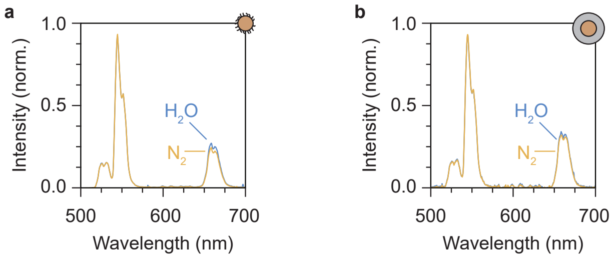

Figure S6 | Upconversion emission spectra of nanocrystal films under different gas glows. (a) Upconversion emission spectrum of $NaYF_4$:Er^{3+}(2%),Yb^{3+}(18%) oleic-acid-coated NCs under a flow of dry nitrogen and a flow containing water vapor (p/p_0 = 0.17). **(b)** Same as **a**, but for a film of silica-coated $NaYF_4$:Er^{3+}(2%),Yb^{3+}(18%).

Section S7: Lifetimes of the upconversion green and red emissions under different gas flows

We describe upconversion luminescence using a simple three-level system. Excitation of a lanthanide ions in the near-infrared with rate k_{exc} builds up a fractional population of ions in an intermediate, near-infrared-emitting level n_{NIR}:

$$\frac{\mathrm{d}n_{\mathrm{NIR}}}{\mathrm{d}t} = +k_{\mathrm{exc}}\left[1 - n_{\mathrm{NIR}}(t)\right] - k_{\mathrm{ET}} n_{\mathrm{NIR}}^2. \tag{6}$$

Upconversion proceeds by ET from these near-infrared-emitting level, with rate k_{ET}. Populations of the green- and red-emitting levels n_{G} and n_{R} are

$$\frac{\mathrm{d}n_{\mathrm{R,G}}}{\mathrm{d}t} = +k_{\mathrm{ET}} n_{\mathrm{NIR}}^2(t) - k_{\mathrm{R,G}} n_{\mathrm{R,G}}, \tag{7}$$

with $k_{\mathrm{R,G}}$ the decay rates of the two levels. The assumption of weak excitation is valid as we estimate $k_{\mathrm{exc}} \approx 10^{-4}$ ms^{-1} (using the absorption cross-section of ytterbium in $NaGdF_4$ 1.1×10^{-21} cm^2 (Ref. S1) and an excitation power of 10 Wcm^{-2})—much slower than even the bulk radiative decay rate of ytterbium (0.58 ms^{-1}; Ref S4). Furthermore, we do not take into account the $NaYF_4$ crystal structure and ignore the effects of cross-relaxation and energy transfer. In the limit of low excitation power (i.e. $k_{\mathrm{exc}} \ll k_{\mathrm{NIR}}$), the steady-state intensity of the green and red emissions $I_{\mathrm{RG}}^{\mathrm{SS}}$ scales as

$$I_{\mathrm{RG}}^{\mathrm{SS}} \propto k_{\mathrm{R,G}} k_{\mathrm{NIR}}^2. \tag{8}$$

We fitted the decay curves in Figure 3d–e (main text) to the model (Eq. S6,S7), assuming the limit of weak excitation and assuming a steady-state population of the green and red-emitting levels after 2 ms of laser excitation. The resulting fit parameters are $k_{\mathrm{NIR}}^{\mathrm{N_2}}$, $k_{\mathrm{NIR}}^{\mathrm{H_2O}}$, $k_{\mathrm{G}}^{\mathrm{N_2}}$, $k_{\mathrm{G}}^{\mathrm{H_2O}}$, $k_{\mathrm{R}}^{\mathrm{N_2}}$ and $k_{\mathrm{R}}^{\mathrm{H_2O}}$ of 15.7, 17.4, 7.0, 7.8, 2.5 and 2.6 ms^{-1}.

For silica-capped NCs (see Fig. S7c–d), the fit parameters are $k_{\mathrm{NIR}}^{\mathrm{N_2}}$, $k_{\mathrm{NIR}}^{\mathrm{H_2O}}$, $k_{\mathrm{G}}^{\mathrm{N_2}}$, $k_{\mathrm{G}}^{\mathrm{H_2O}}$, $k_{\mathrm{R}}^{\mathrm{N_2}}$ and $k_{\mathrm{R}}^{\mathrm{H_2O}}$ of 19.0, 20.0, 3.2, 3.0, 4.3 and 5.2 ms^{-1}.

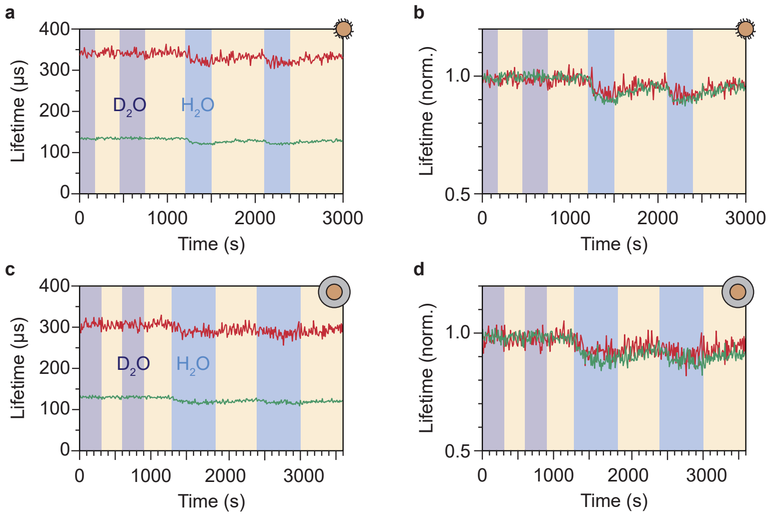

Figure S7 | Upconversion emission lifetimes under different gas glows. **(a)** Upconversion emission lifetimes of $NaYF_4{:}Er^{3+}$(2%),Yb^{3+}(18%) oleic-acid-coated NCs, under changing flows of dry nitrogen and a flow containing water vapor ($p/p_0 = 0.17$; blue shaded areas for H_2O and purple for D_2O). Lifetimes are obtained by fitting a single exponential to decay curves with an integration time of 10 s. **(b)** Same as **a**, but normalized to the lifetime under dry nitrogen flow. **(c–d)** Traces of the green and red photoluminescence of silica-coated $NaYF_4{:}Er^{3+}$(2%),Yb^{3+}(18%) NCs excited at 980 nm under dry nitrogen flow (yellow) and in the presence of water vapor (blue). The laser was operated in block-pulsed mode, where it was on for the first 2 ms (unshaded area) and off for the last 2 ms (shaded area). Solid lines are fits to the rate-equation model (Eq. 7–8). **(e–f)** Same as **a**–**b**, but for a film of silica-coated $NaYF_4{:}Er^{3+}$(2%),Yb^{3+}(18%) NCs.

Section S8: Infrared absorption isotherms of D_2O and H_2O vapor

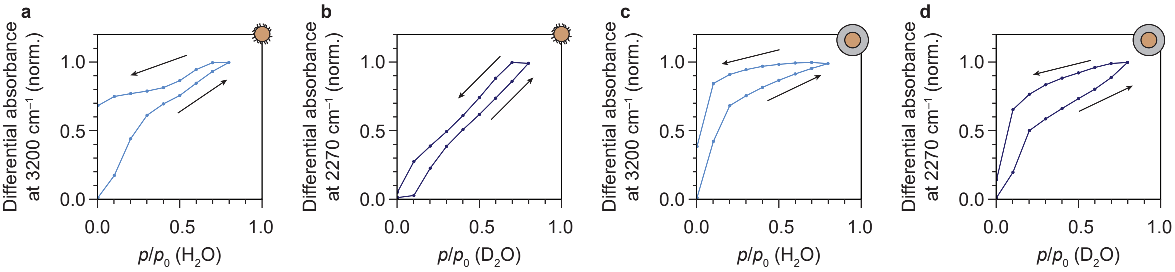

Figure S8 | Adsorption isotherms of water vapor based on ATR-FTIR spectroscopy. **(a)** Differential absorbance at 3200 cm^{-1} as a function of the relative water pressure p/p_0 (H_2O), for a film of $NaYF_4$:Er^{3+}(2%),Yb^{3+}(18%) oleic-acid-coated NCs. Arrows indicate the adsorption and desorption isotherms. **(b)** Same as **a**, but for adsorption/desorption of D_2O vapor. **(c–d)** Same as **a–b**, but for adsorption/desorption of H_2O and D_2O vapor on a film of silica-coated $NaYF_4$:Er^{3+}(2%),Yb^{3+}(18%) NCs.

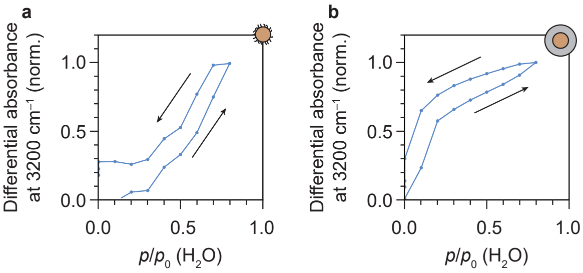

Figure S9 | Equilibrium adsorption isotherms of water vapor based on ATR-FTIR spectroscopy. **(a)** Differential absorbance at 3200 cm^{-1} as a function of the relative water pressure p/p_0 (H_2O), for a film of oleic-acid-coated $NaYF_4$:Er^{3+}(2%),Yb^{3+}(18%) NCs. Arrows indicate the adsorption and desorption isotherms. **(b)** Same as **a**, but for adsorption/desorption of H_2O on a film of silica-coated $NaYF_4$:Er^{3+}(2%),Yb^{3+}(18%) NCs. These isotherms were obtained under equilibrium conditions after prior exposure to water vapor.

Section S9: Exchange experiments with D_2O and H_2O vapor

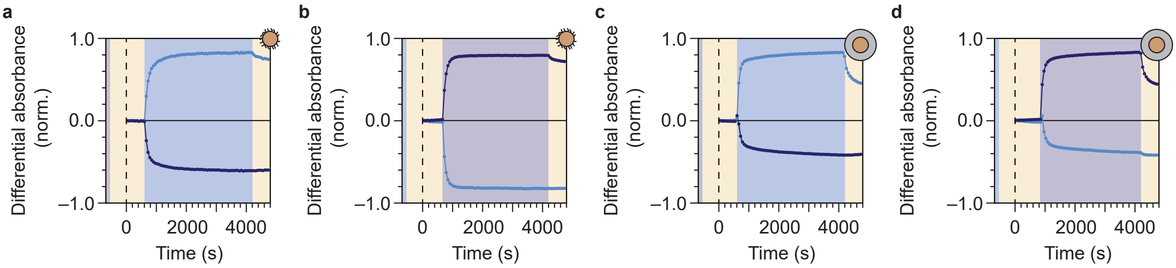

Figure S10 | Exchange experiments with D_2O and H_2O vapor. **(a)** Normalized differential absorbance at 3200 cm^{-1} (blue data points) and 2270 cm^{-1} (purple) for a film of oleic-acid-coated $NaYF_4$:Er^{3+}(2%),Yb^{3+}(18%) NCs, measured under dry nitrogen flow (yellow-shaded areas) or in the presence of H_2O vapor ($p/p_0 = 0.17$). The sample was purged with D_2O vapor before starting the experiment. **(b)** Same as **a**, but for the same film under exposure to D_2O vapor ($p/p_0 = 0.17$) and prior purging with H_2O vapor. **(c–d)** Same as **a–b**, but for a film of silica-coated $NaYF_4$:Er^{3+}(2%),Yb^{3+}(18%) NCs.

Section S10: Background signal on blank ATR crystals

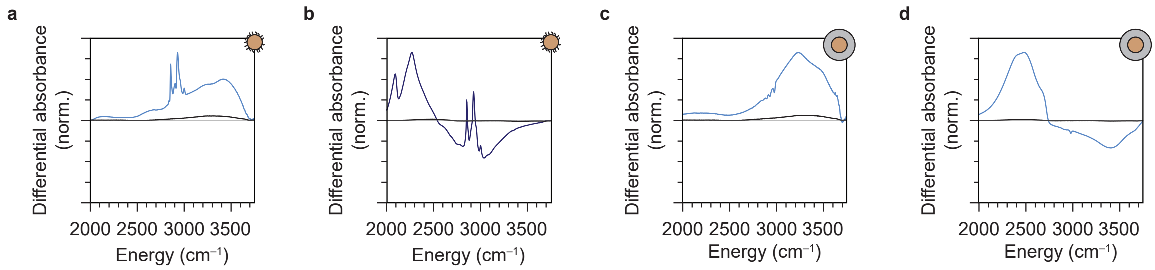

Figure S11. **(a)** Differential infrared absorption spectra of a layer of oleic-acid-coated $NaYF_4$:Er^{3+}(2%),Yb^{3+}(18%) NCs (blue) and on a blank ATR crystal (black), under a flow of $p/p_0 = 0.20$ H_2O. The signal is relative to the infrared absorption under dry nitrogen flow. **(b)** Same, but for a flow of $p/p_0 = 0.20$ D_2O. **(c)** Same, but for a layer of silica-coated $NaYF_4$:Er^{3+}(2%),Yb^{3+}(18%) NCs. **(d)** Same as **c**, but for a flow of $p/p_0 = 0.20$ D_2O.

Supporting References

(S1) Villanueva-Delgado, P.; Biner, D.; Krämer, K.W.; Judd–Ofelt Analysis of β-$NaGdF_4$:Yb^{3+},Tm^{3+} and β-$NaGdF_4$:Er^{3+}, *J. Lumin.* **2017**, *189*, 84–90.

(S2) Carnall, W.T.; Crosswhite, H.; Crosswhite, H.M.; Energy Level Structure and Transition Probabilities in the Spectra of the Trivalent Lanthanides in LaF_3, *Argonne Nat. Lab.; Argonne, IL* **1978**

(S3) Senden, T.; Rabouw, F. T.; Meijerink, A.; Photonic Effects on the Radiative Decay Rate and Luminescence Quantum Yield of Doped Nanocrystals, *ACS Nano* **2015**, *9*, 1801–1808.

(S4) Rabouw, F. T.; Prins, P. T.; Villanueva-Delgado, P.; Castelijns, M.; Geitenbeek, R. G.; Meijerink, A.; Quenching Pathways in $NaYF_4$:Er^{3+},Yb^{3+} Upconversion Nanocrystals, *ACS Nano* **2018**, *12*, 4812–4823.

(S5) van Wijngaarden, J. T.; Scheidelaar, S.; Vlugt, T. J. H.; Reid, M. F.; Meijerink, A.; Energy Transfer Mechanism for Downconversion in the (Pr^{3+}, Yb^{3+}) Couple, *Phys. Rev. B* **2010**, *81*, 155112.

(S6) Yu, D. C.; Rabouw, F. T.; Boon, W. Q.; Kieboom, T., Ye S.; Zhang, Q. Y.; Meijerink, A.; Insights into the Energy Transfer Mechanism in Ce^{3+}-Yb^{3+} Codoped YAG Phosphors, *Phys. Rev. B* **2014**, *90*, 165126.

(S7) Rabouw, F. T.; den Hartog, S. A.; Senden, T.; Meijerink, A.; Photonic Effects on the Förster Resonance Energy Transfer Efficiency, *Nat. Commun.* **2014**, *5*, 3610.

(S8) Aebischer, A; Hostettler, M; Hauser, J.; Krämer, K.; Weber, T.; Güdel, H. U.; Bürgi, H. B.; Structural and Spectroscopic Characterization of Active Sites in a Family of Light-Emitting Sodium Lanthanide Tetrafluorides, *Angew. Chem. Int. Ed.* **2006**, *45*, 2802–2806.

(S9) Himmelstoss, S. F.; Hirsch, T.; Long-Term Colloidal and Chemical Stability in Aqueous Media of $NaYF_4$-Type Upconversion Nanoparticles Modified by Ligand Exchange, *Part. Part. Syst. Charact.* **2019**, *36*, 1900235.

(S10) Coblentz Society, Inc.; NIST Chemistry WebBook, NIST Standard Reference Database Number 69, Evaluated Infrared Reference Spectra, Spectrum ID = C108883, C108941, C67641, C64175, National Institute of Standards and Technology, Gaithersburg MD retrieved May 19th 2023, https://doi.org/10.18434/T4D303.

(S11) van Swieten, T. P.; Yu, D.; Yu, T.; Vonk, S. J. W.; Suta, M.; Zhang, Q.; Meijerink, A.; Rabouw, F. T.; A Ho^{3+}-Based Luminescent Thermometer for Sensitive Sensing over a Wide Temperature Range, *Adv. Optical Mater.* **2021**, *9*, 2001518.
